# Provider Perspectives on Artificial Intelligence–Guided Screening for Low Ejection Fraction in Primary Care: Qualitative Study

**DOI:** 10.2196/41940

**Published:** 2022-10-14

**Authors:** Barbara Barry, Xuan Zhu, Emma Behnken, Jonathan Inselman, Karen Schaepe, Rozalina McCoy, David Rushlow, Peter Noseworthy, Jordan Richardson, Susan Curtis, Richard Sharp, Artika Misra, Abdulla Akfaly, Paul Molling, Matthew Bernard, Xiaoxi Yao

**Affiliations:** 1 Division of Health Care Delivery Research Mayo Clinic Rochester, MN United States; 2 Robert D. and Patricia E. Kern Center for the Science of Health Care Delivery Mayo Clinic Rochester, MN United States; 3 Knowledge and Evaluation Research Unit Mayo Clinic Rochester, MN United States; 4 Department of Quantitative Health Sciences Mayo Clinic Rochester, MN United States; 5 Department of Family Medicine Mayo Clinic Rochester, MN United States; 6 Department of Cardiovascular Medicine Mayo Clinic Rochester, MN United States; 7 Biomedical Ethics Research Program Mayo Clinic Rochester, MN United States; 8 Department of Family Medicine Mayo Clinic Health System Mankato, MN United States; 9 Department of Community Internal Medicine Mayo Clinic Health System Eau Claire, WI United States; 10 Department of Family Medicine Mayo Clinic Health System Onalaska, WI United States

**Keywords:** artificial intelligence, AI, machine learning, human-AI interaction, health informatics, primary care, cardiology, pragmatic clinical trial, AI-enabled clinical decision support, human-computer interaction, health care delivery, clinical decision support, health care, AI tools

## Abstract

**Background:**

The promise of artificial intelligence (AI) to transform health care is threatened by a tangle of challenges that emerge as new AI tools are introduced into clinical practice. AI tools with high accuracy, especially those that detect asymptomatic cases, may be hindered by barriers to adoption. Understanding provider needs and concerns is critical to inform implementation strategies that improve provider buy-in and adoption of AI tools in medicine.

**Objective:**

This study aimed to describe provider perspectives on the adoption of an AI-enabled screening tool in primary care to inform effective integration and sustained use.

**Methods:**

A qualitative study was conducted between December 2019 and February 2020 as part of a pragmatic randomized controlled trial at a large academic medical center in the United States. In all, 29 primary care providers were purposively sampled using a positive deviance approach for participation in semistructured focus groups after their use of the AI tool in the randomized controlled trial was complete. Focus group data were analyzed using a grounded theory approach; iterative analysis was conducted to identify codes and themes, which were synthesized into findings.

**Results:**

Our findings revealed that providers understood the purpose and functionality of the AI tool and saw potential value for more accurate and faster diagnoses. However, successful adoption into routine patient care requires the smooth integration of the tool with clinical decision-making and existing workflow to address provider needs and preferences during implementation. To fulfill the AI tool’s promise of clinical value, providers identified areas for improvement including integration with clinical decision-making, cost-effectiveness and resource allocation, provider training, workflow integration, care pathway coordination, and provider-patient communication.

**Conclusions:**

The implementation of AI-enabled tools in medicine can benefit from sensitivity to the nuanced context of care and provider needs to enable the useful adoption of AI tools at the point of care.

**Trial Registration:**

ClinicalTrials.gov NCT04000087; https://clinicaltrials.gov/ct2/show/NCT04000087

## Introduction

Advances in artificial intelligence (AI) that are poised to transform health care are hindered by implementation challenges [1,2] that call for attention to provider needs and user-centeredness [3,4]. As AI models are increasingly pushed to the point of care, front-line care teams are often left to solve the challenges of AI integration on their own [5]. Research is needed to ensure the clinical value of AI tools is preserved through successful adoption at the point of care. To inform this knowledge gap, we present a case study of a pragmatic trial in which an AI-enabled screening tool was introduced in primary care to help identify patients with a high likelihood of unrecognized left ventricular low ejection fraction (EF) [6]. Low EF is often underdiagnosed but treatable; early diagnosis and treatment could prevent the progression of heart failure and reduce future hospitalization and mortality. We offer a qualitative analysis of provider reflections on the use of the AI screening tool and suggestions for the effective clinical adoption of AI-enabled tools.

## Methods

### Overall Study Design

**Figure 1:**
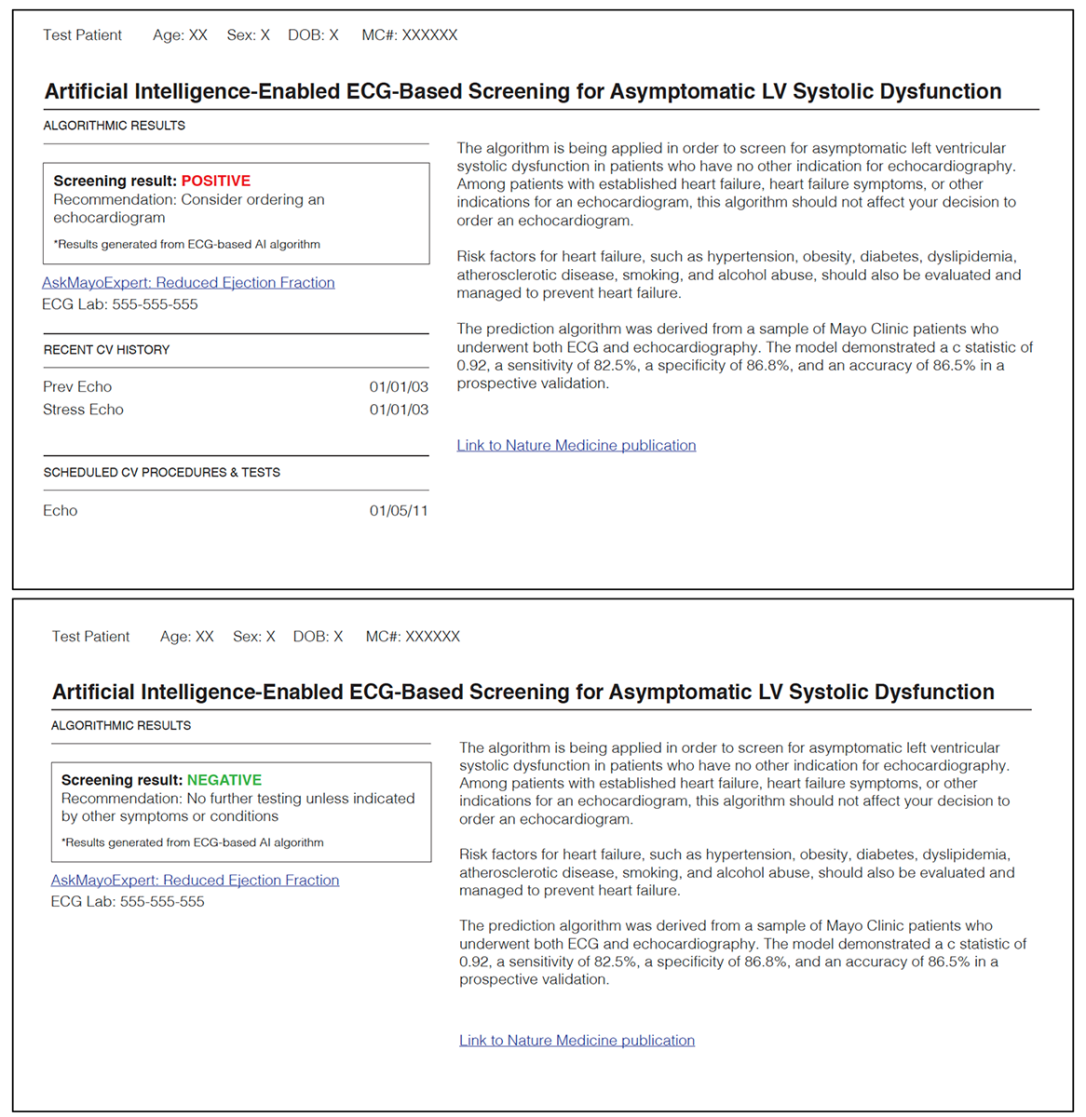
Example AI result report. AI reports are generated by the AI tool and embedded into the electronic health record. Note that only positive results would generate an email to a provider, and both positive and negative results could be accessed in the patient’s health record. AI: artificial intelligence; ECG: electrocardiogram; LV: left ventricular.

A pragmatic cluster randomized controlled trial (NCT04000087) was conducted to evaluate whether an electrocardiogram (ECG) AI-guided screening tool (ECG AI-Guided Screening for Low Ejection Fraction; EAGLE) improves the diagnosis of left ventricular EF in clinical practice [7,8]. Details on the trial design are reported elsewhere [6]. The intervention is a provider-facing action-recommendation report (Figure 1) that contains a screening result generated by the application of a deep learning algorithm to a patient’s ECG [9].

Positive screening results were delivered to providers via an email alert that suggests a transthoracic echocardiogram (TTE) should be considered and remind them that the report was available in the electronic health record (EHR). The report included a brief description of the AI algorithm and a phone number to call for additional information. Follow-up emails were sent if no TTE was ordered or no rationale was provided for rejecting the TTE recommendation.

### Focus Group Study Design and Procedures

Semistructured focus groups were conducted with 10 primary care teams. We used a positive deviance approach to select the care teams [10,11]. Specifically, we selected the 5 care teams with the lowest TTE recommendation adherence and the 5 teams with highest adherence, with adherence defined as acting on the AI recommendation by ordering a TTE. Each focus group was conducted with providers from the same care team. Individual interviews were conducted to accommodate provider schedules when necessary. Discussion topics included provider experiences with the AI tool and their attitudes toward AI in medicine. Between December 2019 and February 2020, a total of 7 focus groups and 5 individual interviews were conducted, involving 29 providers consisting of physicians, physician assistants, and nurse practitioners. Participant characteristics are summarized in Table 1. The 2 interviewers and all interviewees were blinded to the adherence status of the care team to enable candid, nondefensive conversation as well as to avoid biasing the interviewers [12]. All focus groups were audio recorded, transcribed verbatim, deidentified, and reviewed for accuracy.

**Table 1:** Characteristics of focus group participants in high- and low-adherence care teams. Note that characteristic information from 2 participants is missing.

Characteristic			High adherence (N=17)		Low adherence (N=10)
**Age (years)**					
	n	15		10	
	Mean (SD)	44.8 (9.07)		41.4 (3.92)	
	Median	46.0		41.0	
	Range	32.0-61.0		36.0-50.0	
**Gender (self-reported), n (%)**					
	Male	8 (47)		6 (60)	
	Female	9 (53)		4 (40)	
**Race, n (%)**					
	White	16 (94)		8 (80)	
	Other^a^	0 (0)		1 (10)	
	Prefer not to say	1 (6)		1 (10)	
**Position, n (%)**					
	Physician	12 (71)		8 (80)	
	Physician assistant	0 (0)		2 (20)	
	Nurse practitioner	5 (29)		0 (0)	
**Specialty, n (%)**					
	Family medicine	11 (65)		5 (50)	
	Internal medicine	6 (35)		5 (50)	
**Years in practice**					
	n	15		10	
	Mean (SD)	13.5 (9.04)		8.3 (5.19)	
	Median	11.0		5.5	
	Range	1.0-31.0		3.0-20.0	
**Years in current care team**					
	n	15		10	
	Mean (SD)	11.5 (9.08)		7.2 (6.00)	
	Median	11.0		5.0	
	Range	0.5-31.0		1.0-20.0	

^a^Racial categories measured included American Indian or Alaskan Native, Asian, Black or African American, and Native Hawaiian or Other Pacific Islander. None of our participants identified as being in these categories.

### Data Analysis

Thematic analysis was used to identify predominant themes regarding clinicians’ experiences and perspectives regarding using the AI screening tool [13-15]. Two researchers (BB and XZ) open-coded transcripts and then categorized open codes into themes. The relationships between the themes were then articulated in a hierarchical structure of main themes and subthemes. The thematic structure was revised when new categories and themes were identified. Analytic memos were used to summarize the findings. NVivo software (version 12; QSR International) was used to facilitate analysis. Researchers were unaware of the adherence status of the care teams during coding. Adherence status was revealed to the researchers after all transcripts were coded to assess differences between groups.

### Ethics Approval

The methods were performed in accordance with the relevant guidelines and regulations and approved by the Mayo Clinic Institutional Review Board (IRB #19-003137). The trial was registered on ClinicalTrials.gov (NCT04000087) on June 27, 2019.

## Results

### Perspectives and Themes

All providers received at least one positive AI screening result and were able to correctly describe the AI tool’s functionality and purpose. Providers had polarized perspectives on the value of the AI tool: some expressed that the tool could improve patient care, whereas others thought it was unnecessary or costly. Dissatisfied providers agreed that honing the tool and its delivery would increase value, whereas a small number of providers disagreed with the need for the tool.

We did not observe prominent differences in themes between care teams with high and low adherence. We identified 7 dominant themes of provider reflections on AI tool use: (1) promising clinical value, (2) integration with clinical reasoning, (3) cost-effectiveness and resource allocation, (4) provider training, (5) workflow integration, (6) care pathway coordination, and (7) provider-patient communication.

### Promising Clinical Value

Providers believed in the AI tool’s capability to identify asymptomatic patients at risk for heart failure. Providers saw an opportunity to accelerate care for patients who might otherwise fail to report symptoms of low EF and saw value in implementing early management to save patients from acute cardiac events. Providers also noted the ability of the AI tool to make care more efficient by assessing the ECG more quickly than a provider could.

[The result] was definitely abnormal, and I was able to talk with this patient about lifestyle changes and actually have something coming behind me within that.

Focus group #12

I’m still pushing the button on the order cuz I agree with it but, you know, doing all the nuts and bolts behind it, if that’s done for me, then I can focus my time on doing what only I can do.

Focus group #9

### Integration With Clinical Reasoning

Providers expressed apprehension about the utility and long-term patient benefit of the tool based on how it fit into clinical practice during the trial. They were concerned about the increased burden, especially when a screening was not clinically useful in patient contexts such as preexisting cardiovascular conditions, and noted that for certain patients, other medical priorities (eg, cancer treatment) might take precedence over initiating a TTE and treatment for low EF. Providers expressed concern regarding the lack of clear guidelines about when to order a new TTE if there were prior TTEs in a patient’s medical record. A few providers were unsure to what extent the AI tool could improve patients’ long-term health outcomes and noted barriers treating a patient who may be at risk for heart failure but has not yet shown any symptoms, revealing a potential lack of knowledge about evidence-based recommendations for asymptomatic low EF treatment.

They had known heart disease. I was like, “Well, that doesn't make any sense.” After the first couple of doing that, I started almost disregarding.

Focus group #10

I only had three, and I know them. I knew them very well, so the minute I got the one with the end-stage liver failure, cirrhosis, paracentesis, I knew that immediately that that wasn’t gonna be valid, or not necessarily not valid, but is it correct?

Focus group #12

Providers gauged the AI’s capability relative to their own. Some providers believed that the AI tool was superior in recognizing patterns to identify asymptomatic cases. A few others preferred face-to-face visits for physical examination and continuity of care. Some providers were also concerned a bias might occur if the AI algorithm was trained on data misaligned with their patient panels.

Good I got a notification. I woulda missed it [the diagnosis].

Focus group #10

Uh, we can do a lot by remote monitoring, but I need to touch you, and I need to listen to you, and I need to listen to your heart. And so if something just got triggered, I don’t care.

Focus group #7

The struggle with AI so far has been that the breakdowns have come because of the data that’s been input and a lot of that has been because of our geographic or our social or our...

Interviewee #5

There’s bias.

Interviewee #3; focus group #1

### Cost-effectiveness and Resource Allocation

Some providers questioned the cost-effectiveness of the recommended TTE follow-up given the current lack of outcome data and noted that the cost is especially concerning when the screening result is a false positive. A few clinicians were concerned about insurance coverage. Some clinicians noted potential cumulative cost savings from optimized treatment plans and the prevention of heart failure hospitalizations. However, providers noted that increased TTE order volume due to positive AI screenings could delay care for patients with more urgent TTE needs.

You can tell them, thankfully, it’s normal. Obviously, the EKG picked up something that showed potential for concern. We have good news that everything is normal. We’re gonna continue to optimize your treatment. That being said, it’s several thousand dollars.

Focus group #8

### Provider Training

Providers remembered being introduced to the AI tool and trial protocol by department leadership in meetings and via email yet did not recall the information when they received the AI result. Providers reported agreeing, sometimes enthusiastically, with the objectives of the trial but found it difficult to translate the instructions (eg, ordering TTE based on AI result) into their context of care. Championship by leadership set unintended high expectations for the AI tool and caused disappointment when the number of positive screenings was lower than expected. Providers also remarked that they could not remember how to find the AI report in the EHR and did not have time to read the training packet.

Right. I mean, the first email inviting us, I read two paragraphs. “Boy, that sounds like a good idea.” Then all of a sudden, we get this book, and then all of a sudden, they [AI results] start coming. You just get lost.

Focus group #10

No. We didn’t see the video.

Interviewee #3

Right there, if it requires a video and a half hour lecture to figure out where [to find the report], then it’s probably not well-placed.

Interviewee #1; focus group #1

### Workflow Integration

Providers were unaware that the delivery of the AI result via email notification outside of the EHR was due to system security issues, Food and Drug Administration regulations, and IT barriers, which fragmented the digital workflow. The AI report in the EHR was rarely accessed by providers after the receipt of the AI result email notification. Repeated reminder emails urging providers to act on unresolved AI results were irritating and created confusion about which alerts had been completed and which needed attention. The AI result delivery was not timed to be part of a scheduled visit, which caused extra clerical and cognitive burden, and took time away from providers’ already busy schedules.

There was an ECG that suggests you might do an echo and if I’m with the patient right there, done, but to [Interviewee’s] point, it was when it was noncontiguous, non-need. It was an extra half an hour phone call in the day that I just simply don’t have time for.

Focus group #1

I got emails, which made it very difficult because it’s not linked to the chart.

Focus group #3

### Care Pathway Coordination

The AI report was always routed to the patient’s primary care provider regardless of why and by whom the ECG was ordered, causing confusion among primary care providers about care coordination and the chain of custody. In cases where the AI result email alerts were from ECGs ordered outside of primary care (eg, in the emergency department), the primary care providers questioned whether these AI results were within the scope of their responsibility. Some providers felt they were caught between the care pathway already underway (eg, for surgery) and a potentially new or redundant care pathway suggested by the AI result. They felt that they were stuck in an awkward position, either ignoring the alert or communicating a result to a colleague who would have already been aware. In these cases, the AI tool was seen as not being logically coordinated within the care pathway.

Right now he’s in the hospital, and Cardiology’s definitely onboard, and so I just gave them that heads-up.

Focus group #2

The EAGLE thing triggers to us. We don’t know whether we are supposed to follow up and do everything. I don’t know whether I ordered [the ECG].

Focus group #6

I'm a minutiae guy, so if someone’s got an abnormal EKG, I look at their EKG. I look at their echo. It puts a fair amount of burden back to the PCP because no matter where it’s ordered, it comes back to me as PCP.

Focus group #8

### Provider-Patient Communication

Some providers stated that the unexpected nature of a result generated outside the context of a visit, the lack of explainability of deep learning, and the lack of reporting guidelines regarding false positives make communicating the results to patients challenging and time-consuming. In a worst-case scenario for patient-provider alliance and provider morale, a patient perceived that AI corrected an error made by the provider. Providers disagreed on whether patients can understand and cope with the AI results if the results are automatically delivered to patients without provider oversight and communication of the results. Some providers considered the AI tool new and complex, whereas others considered it similar to screenings that patients already view as routine (eg, a blood test).

You have to explain to the patient what you’re gonna do when you get the low EF; “how come you didn’t figure it out already, Dr. [Name]; if you’re such a great clinician, how could you miss this?”

Focus group #1

But in terms of just calling somebody up out of the...on just like a cold call and saying, “I think you might have heart failure” because a computer said so, um, that’s where my caveats come from.

Focus group #7

### Provider Suggestions to Improve Future AI Tool Adoption

Providers articulated the following suggestions to improve future AI tool uptake and use: (1) setting appropriate expectations for how, when, and how often the AI tool would deliver a recommendation; (2) attuning the application of the AI tool to patient populations; (3) having reliable data that show positive clinical outcomes due to the tool; (4) having a demonstration of cost-effectiveness, (5) streamlining integration into clinical workflow, (6) clarifying provider responsibility, and (7) having support for the communication of results to patients.

## Discussion

### Principal Findings

We found that most providers saw the potential value of the AI tool for more accurate and faster diagnoses. They were willing to adopt such tools and collaborate with researchers to validate tools in clinical practice. However, during use in the clinical trial, providers identified challenges that should be taken into consideration as AI tools are introduced widely in primary care. Provider recommendations encompassed increased sensitivity to clinical decision-making, addressing digital implementation issues, and the awareness of system-wide impact.

AI tools that predict asymptomatic health conditions convoke a set of issues in medical decision-making that providers are asked to resolve on a case-by-case basis and, in doing so, are confronted with a change in the scope of their clinical decision-making [16]. Although AI tools provide guidance, they rarely apply to all patients and often add a new dimension to already complex decision-making [17,18]. In practical use, providers have different ways of weighing evidence to inform the best next step in patient care. For example, although the confirmation or rejection of an AI screening result through a follow-up testing may seem low-risk and easy, the clinical action in an individual patient’s case could shift focus from a more immediate threat to health and increase cost. In primary care, providers are positioned to see the entire context of care and together with patients navigate multiple risk-benefit decisions within complex situations that do not lend themselves to rapid, binary decisions for next steps [19]. Consequently, the incorporation of AI tools that support new diagnoses can further complicate the issues of distinguishing between the art and science of medicine in complex primary care decision-making [20]. Our research reaffirms that providers may find AI-enabled tools capable of delivering helpful information but that communication and actions taken by the care team in response to AI tools are complex and demand a balance between structured guidance and freedom to adapt information to a clinical case [21,22].

Providers offered suggestions for improving the applicability of the AI model, digital workflow, and patient communication. These suggestions can enhance AI tool use but may be difficult to achieve during the initial translation in a pragmatic clinical trial. Provider feedback to hone the AI model and digital workflow are necessary to ensure the best diagnostic performance over time, safety, and adherence to regulatory requirements. Additional burden on providers during the initial translation may exacerbate clerical burden, which can dampen interest in AI tool adoption. It is important to set expectations with providers that clumsy workarounds and added burden during initial translation in the clinical trial are temporary and that fine-tuning AI implementation to meet various clinical contexts and provider needs is a long-term, collaborative process [23]. Additionally, the silent testing of the AI tool before broader launch in a randomized controlled trial and more spontaneous, passive modes of collecting provider feedback (versus repeatedly requesting active input from providers) may be of value. Moreover, AI tools may illuminate existing issues in care delivery or cause new problems in new contexts, which prompts the need for real-time observations and auditing of AI models and tools to improve the design of the full implementation and enable effective use [24,25].

### Study Limitations

Focus groups were conducted after trial completion, and thus, provider experience was communicated retrospectively. Future research could make use of more spontaneous data collection methods (eg, ecological momentary assessment) to capture provider experiences and perspectives at the point of care in real time. Our findings were based on the perspectives of 29 providers from 10 care teams that may not be representative of the primary care provider population and thus cannot capture the full scope of diverse perspectives among primary care providers. Additionally, it is unclear how our results will generalize beyond AI tools that use a deep learning algorithm and leverage knowledge from cardiology within a primary care setting. Future research with a broader range of AI tools in different clinical settings and specialties with more diverse provider samples is needed to triangulate our findings and uncover additional important themes.

### Conclusion

Our work identified specific issues that providers faced when AI-enabled tools are introduced into primary care during a clinical trial as well as relevant techniques across algorithm development, point-of-care use, and broader systems that can drive the provider-centered adoption of AI tools. These findings corroborate the challenges of implementing AI-enabled tools in medicine: successful implementation must be sensitive to the nuanced context of care and provider sensibilities to enable the useful adoption of AI tools at the point of care.

## Abbreviations

AI: artificial intelligence
EAGLE: ECG AI-Guided Screening for Low Ejection Fraction
ECG: electrocardiogram
EF: ejection fraction
EHR: electronic health record
TTE: transthoracic echocardiogram
